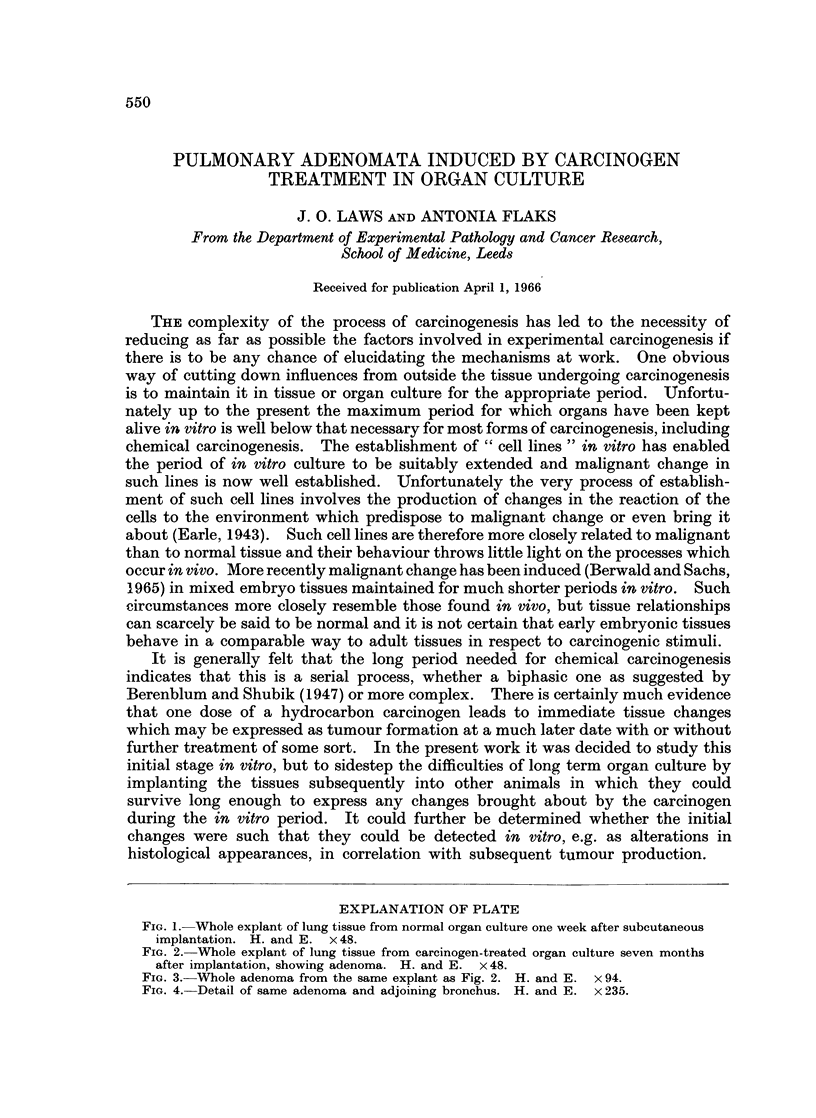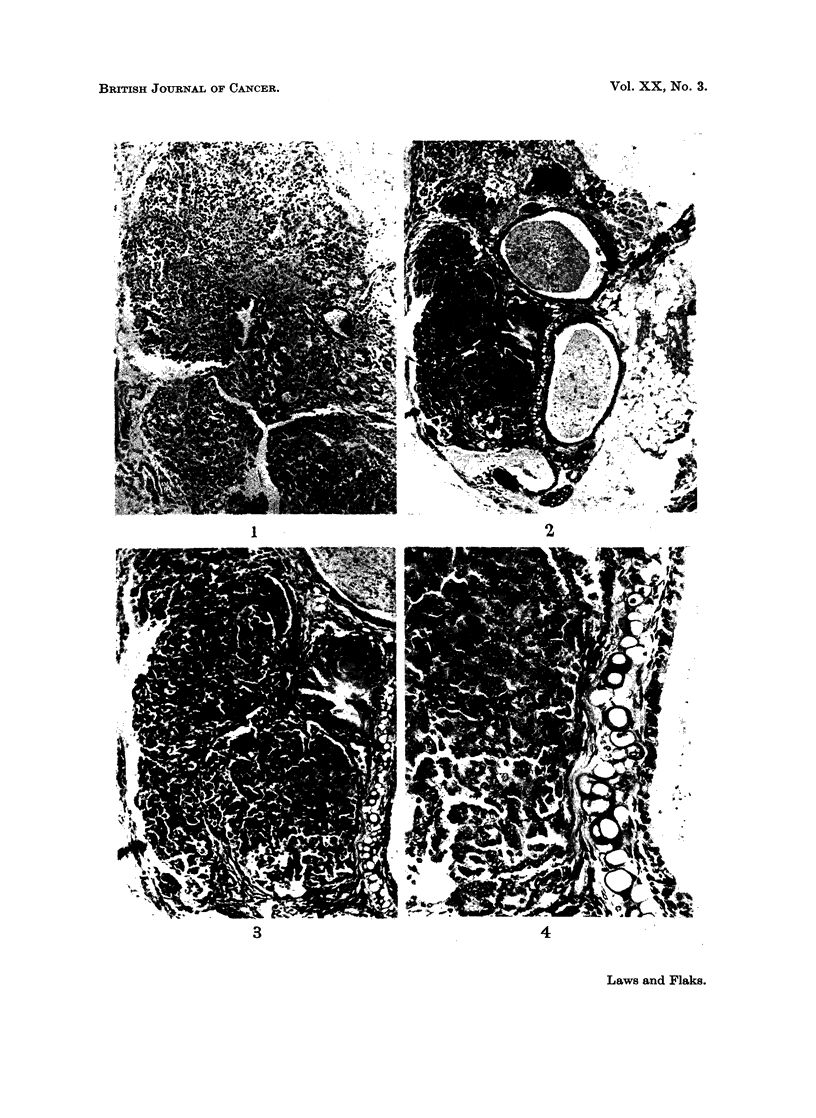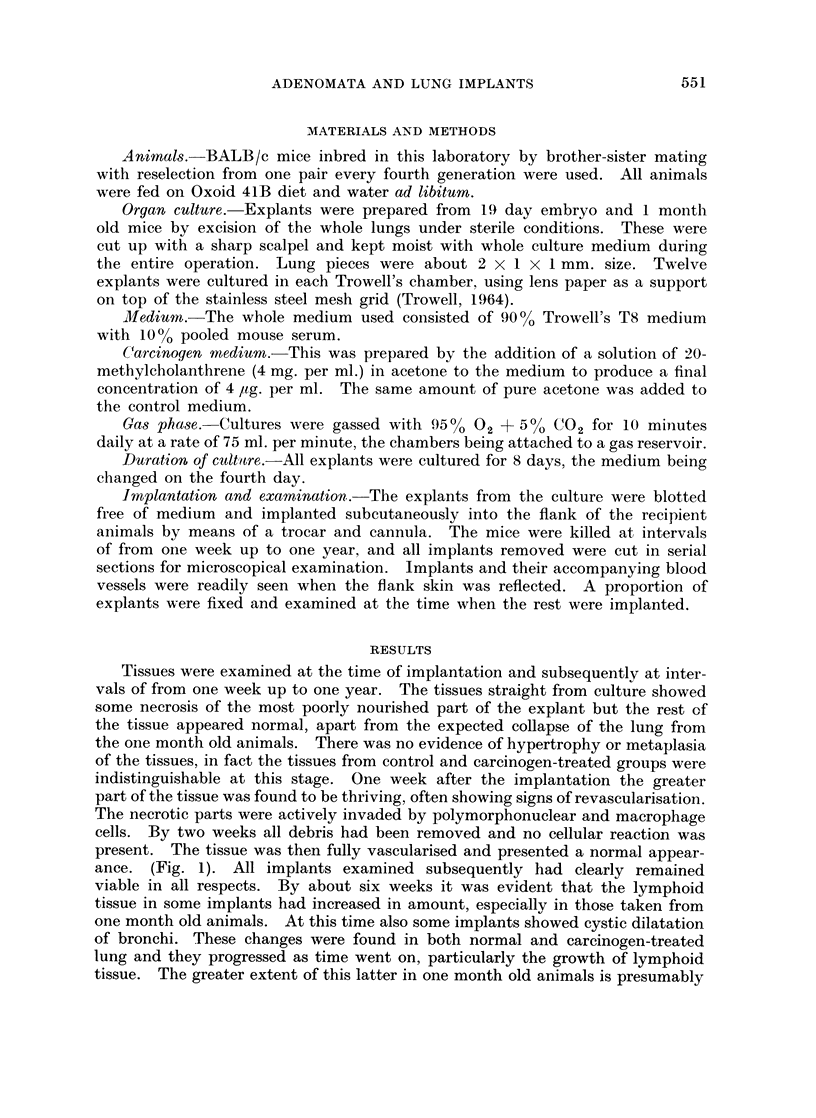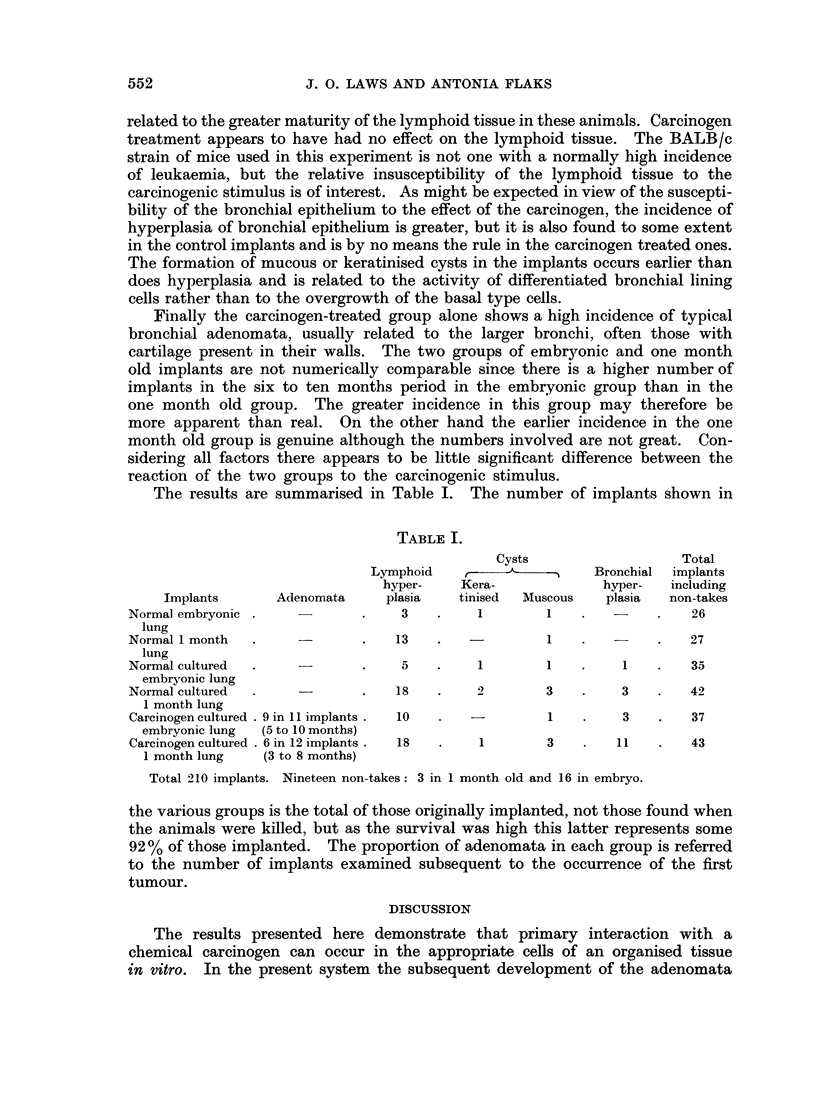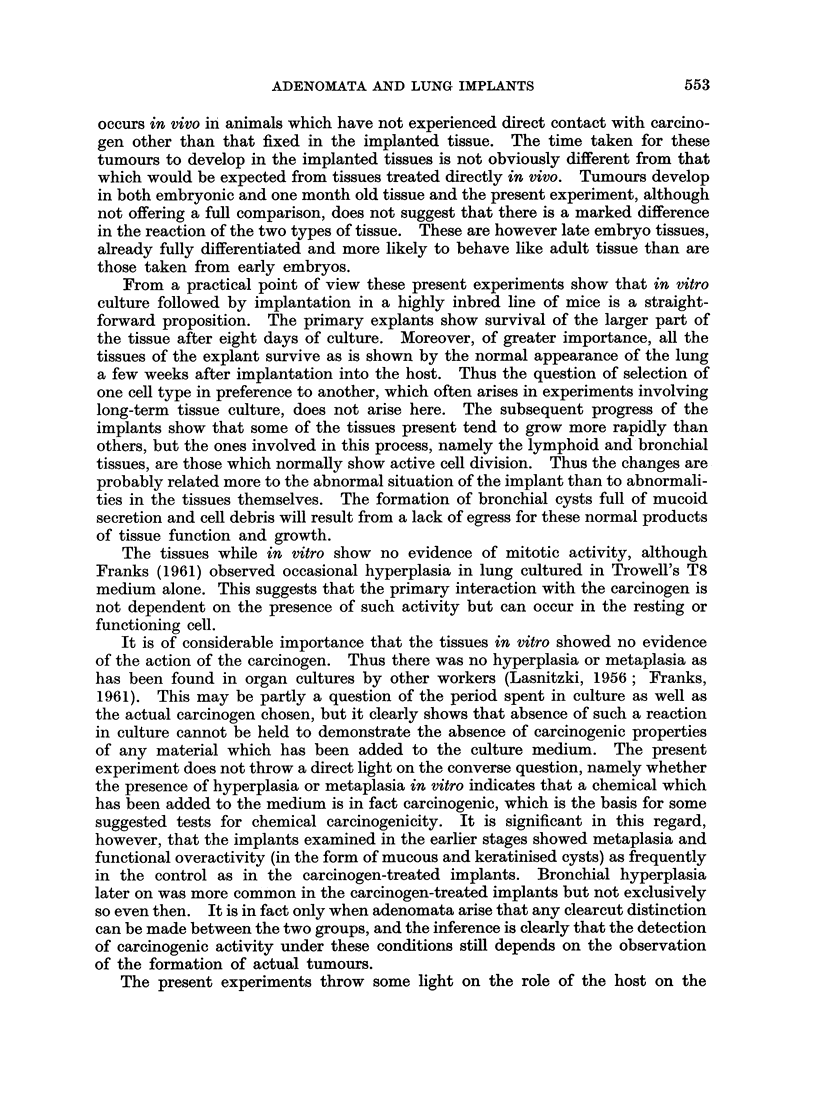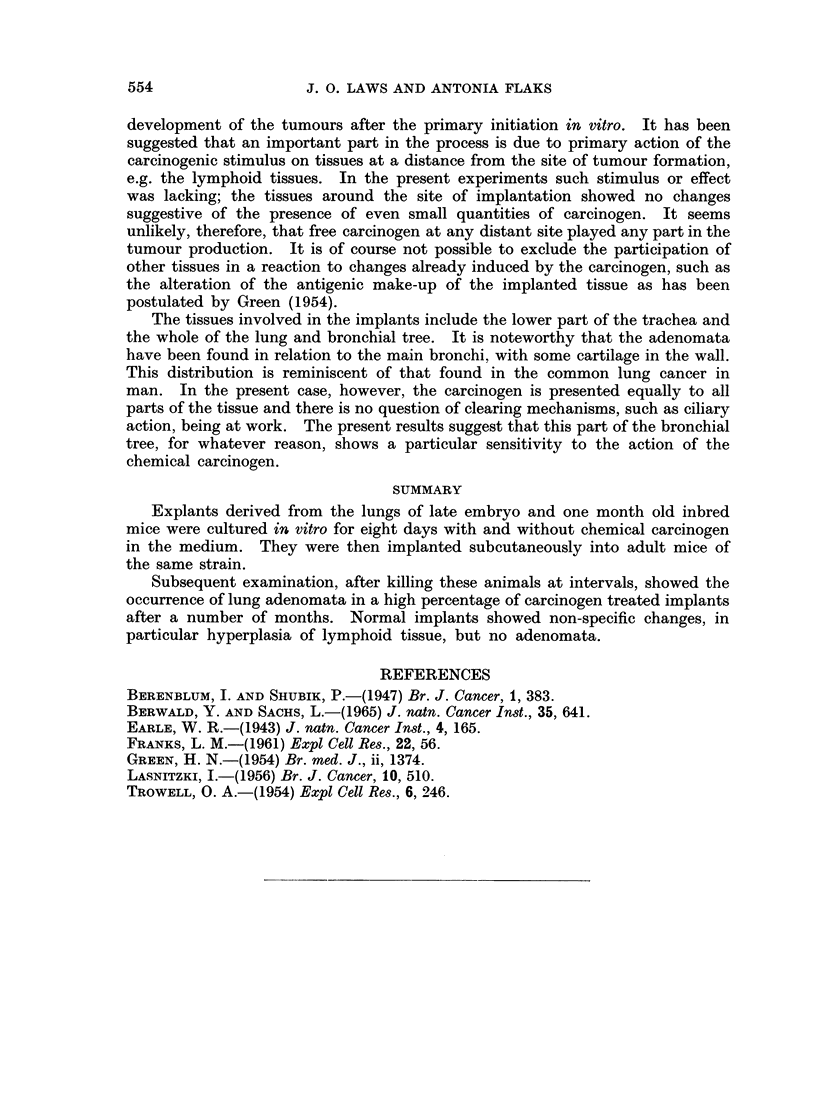# Pulmonary adenomata induced by carcinogen treatment in organ culture.

**DOI:** 10.1038/bjc.1966.68

**Published:** 1966-09

**Authors:** J. O. Laws, A. Flaks

## Abstract

**Images:**


### PULMONARY ADENOMATA INDUCED BY CARCINOGEN TREATMENT IN ORGAN CULTURE

J. O. LAWS AND ANTONIA FLAKS

*From the Department of Experimental Pathology and Cancer Research, School of Medicine, Leeds*

Received for publication April 1, 1966

THE complexity of the process of carcinogenesis has led to the necessity of reducing as far as possible the factors involved in experimental carcinogenesis if there is to be any chance of elucidating the mechanisms at work. One obvious way of cutting down influences from outside the tissue undergoing carcinogenesis is to maintain it in tissue or organ culture for the appropriate period. Unfortunately up to the present the maximum period for which organs have been kept alive *in vitro* is well below that necessary for most forms of carcinogenesis, including chemical carcinogenesis. The establishment of " cell lines " *in vitro* has enabled the period of *in vitro* culture to be suitably extended and malignant change in such lines is now well established. Unfortunately the very process of establishment of such cell lines involves the production of changes in the reaction of the cells to the environment which predispose to malignant change or even bring it about (Earle, 1943). Such cell lines are therefore more closely related to malignant than to normal tissue and their behaviour throws little light on the processes which occur *in vivo*. More recently malignant change has been induced (Berwald and Sachs, 1965) in mixed embryo tissues maintained for much shorter periods *in vitro*. Such circumstances more closely resemble those found *in vivo*, but tissue relationships can scarcely be said to be normal and it is not certain that early embryonic tissues behave in a comparable way to adult tissues in respect to carcinogenic stimuli.

It is generally felt that the long period needed for chemical carcinogenesis indicates that this is a serial process, whether a biphasic one as suggested by Berenblum and Shubik (1947) or more complex. There is certainly much evidence that one dose of a hydrocarbon carcinogen leads to immediate tissue changes which may be expressed as tumour formation at a much later date with or without further treatment of some sort. In the present work it was decided to study this initial stage *in vitro*, but to sidestep the difficulties of long term organ culture by implanting the tissues subsequently into other animals in which they could survive long enough to express any changes brought about by the carcinogen during the *in vitro* period. It could further be determined whether the initial changes were such that they could be detected *in vitro*, e.g. as alterations in histological appearances, in correlation with subsequent tumour production.

---

EXPLANATION OF PLATE

FIG. 1.—Whole explant of lung tissue from normal organ culture one week after subcutaneous implantation. H. and E. ×48.
FIG. 2.—Whole explant of lung tissue from carcinogen-treated organ culture seven months after implantation, showing adenoma. H. and E. ×48.
FIG. 3.—Whole adenoma from the same explant as Fig. 2. H. and E. ×94.
FIG. 4.—Detail of same adenoma and adjoining bronchus. H. and E. ×235.

1

2

3

4

Laws and Flaks.

### MATERIALS AND METHODS

*Animals.*—BALB/c mice inbred in this laboratory by brother-sister mating with reselection from one pair every fourth generation were used. All animals were fed on Oxoid 41B diet and water *ad libitum.*

*Organ culture.*—Explants were prepared from 19 day embryo and 1 month old mice by excision of the whole lungs under sterile conditions. These were cut up with a sharp scalpel and kept moist with whole culture medium during the entire operation. Lung pieces were about $2 \times 1 \times 1$ mm. size. Twelve explants were cultured in each Trowell's chamber, using lens paper as a support on top of the stainless steel mesh grid (Trowell, 1964).

*Medium.*—The whole medium used consisted of 90% Trowell's T8 medium with 10% pooled mouse serum.

*Carcinogen medium.*—This was prepared by the addition of a solution of 20-methylcholanthrene (4 mg. per ml.) in acetone to the medium to produce a final concentration of 4 $\mu$g. per ml. The same amount of pure acetone was added to the control medium.

*Gas phase.*—Cultures were gassed with 95% $O_2$ + 5% $CO_2$ for 10 minutes daily at a rate of 75 ml. per minute, the chambers being attached to a gas reservoir.

*Duration of culture.*—All explants were cultured for 8 days, the medium being changed on the fourth day.

*Implantation and examination.*—The explants from the culture were blotted free of medium and implanted subcutaneously into the flank of the recipient animals by means of a trocar and cannula. The mice were killed at intervals of from one week up to one year, and all implants removed were cut in serial sections for microscopical examination. Implants and their accompanying blood vessels were readily seen when the flank skin was reflected. A proportion of explants were fixed and examined at the time when the rest were implanted.

### RESULTS

Tissues were examined at the time of implantation and subsequently at intervals of from one week up to one year. The tissues straight from culture showed some necrosis of the most poorly nourished part of the explant but the rest of the tissue appeared normal, apart from the expected collapse of the lung from the one month old animals. There was no evidence of hypertrophy or metaplasia of the tissues, in fact the tissues from control and carcinogen-treated groups were indistinguishable at this stage. One week after the implantation the greater part of the tissue was found to be thriving, often showing signs of revascularisation. The necrotic parts were actively invaded by polymorphonuclear and macrophage cells. By two weeks all debris had been removed and no cellular reaction was present. The tissue was then fully vascularised and presented a normal appearance. (Fig. 1). All implants examined subsequently had clearly remained viable in all respects. By about six weeks it was evident that the lymphoid tissue in some implants had increased in amount, especially in those taken from one month old animals. At this time also some implants showed cystic dilatation of bronchi. These changes were found in both normal and carcinogen-treated lung and they progressed as time went on, particularly the growth of lymphoid tissue. The greater extent of this latter in one month old animals is presumably related to the greater maturity of the lymphoid tissue in these animals. Carcinogen treatment appears to have had no effect on the lymphoid tissue. The BALB/c strain of mice used in this experiment is not one with a normally high incidence of leukaemia, but the relative insusceptibility of the lymphoid tissue to the carcinogenic stimulus is of interest. As might be expected in view of the susceptibility of the bronchial epithelium to the effect of the carcinogen, the incidence of hyperplasia of bronchial epithelium is greater, but it is also found to some extent in the control implants and is by no means the rule in the carcinogen treated ones. The formation of mucous or keratinised cysts in the implants occurs earlier than does hyperplasia and is related to the activity of differentiated bronchial lining cells rather than to the overgrowth of the basal type cells.

Finally the carcinogen-treated group alone shows a high incidence of typical bronchial adenomata, usually related to the larger bronchi, often those with cartilage present in their walls. The two groups of embryonic and one month old implants are not numerically comparable since there is a higher number of implants in the six to ten months period in the embryonic group than in the one month old group. The greater incidence in this group may therefore be more apparent than real. On the other hand the earlier incidence in the one month old group is genuine although the numbers involved are not great. Considering all factors there appears to be little significant difference between the reaction of the two groups to the carcinogenic stimulus.

The results are summarised in Table I. The number of implants shown in

TABLE I.

| Implants | Adenomata | Lymphoid hyper-plasia | Cysts: Kera-tinised | Cysts: Muscous | Bronchial hyper-plasia | Total implants including non-takes |
|---|---|---|---|---|---|---|
| Normal embryonic lung | — | 3 | 1 | 1 | — | 26 |
| Normal 1 month lung | — | 13 | — | 1 | — | 27 |
| Normal cultured embryonic lung | — | 5 | 1 | 1 | 1 | 35 |
| Normal cultured 1 month lung | — | 18 | 2 | 3 | 3 | 42 |
| Carcinogen cultured embryonic lung | 9 in 11 implants (5 to 10 months) | 10 | — | 1 | 3 | 37 |
| Carcinogen cultured 1 month lung | 6 in 12 implants (3 to 8 months) | 18 | 1 | 3 | 11 | 43 |

Total 210 implants. Nineteen non-takes: 3 in 1 month old and 16 in embryo.

the various groups is the total of those originally implanted, not those found when the animals were killed, but as the survival was high this latter represents some 92% of those implanted. The proportion of adenomata in each group is referred to the number of implants examined subsequent to the occurrence of the first tumour.

### DISCUSSION

The results presented here demonstrate that primary interaction with a chemical carcinogen can occur in the appropriate cells of an organised tissue *in vitro*. In the present system the subsequent development of the adenomata occurs *in vivo* in animals which have not experienced direct contact with carcinogen other than that fixed in the implanted tissue. The time taken for these tumours to develop in the implanted tissues is not obviously different from that which would be expected from tissues treated directly *in vivo*. Tumours develop in both embryonic and one month old tissue and the present experiment, although not offering a full comparison, does not suggest that there is a marked difference in the reaction of the two types of tissue. These are however late embryo tissues, already fully differentiated and more likely to behave like adult tissue than are those taken from early embryos.

From a practical point of view these present experiments show that *in vitro* culture followed by implantation in a highly inbred line of mice is a straightforward proposition. The primary explants show survival of the larger part of the tissue after eight days of culture. Moreover, of greater importance, all the tissues of the explant survive as is shown by the normal appearance of the lung a few weeks after implantation into the host. Thus the question of selection of one cell type in preference to another, which often arises in experiments involving long-term tissue culture, does not arise here. The subsequent progress of the implants show that some of the tissues present tend to grow more rapidly than others, but the ones involved in this process, namely the lymphoid and bronchial tissues, are those which normally show active cell division. Thus the changes are probably related more to the abnormal situation of the implant than to abnormalities in the tissues themselves. The formation of bronchial cysts full of mucoid secretion and cell debris will result from a lack of egress for these normal products of tissue function and growth.

The tissues while *in vitro* show no evidence of mitotic activity, although Franks (1961) observed occasional hyperplasia in lung cultured in Trowell's T8 medium alone. This suggests that the primary interaction with the carcinogen is not dependent on the presence of such activity but can occur in the resting or functioning cell.

It is of considerable importance that the tissues *in vitro* showed no evidence of the action of the carcinogen. Thus there was no hyperplasia or metaplasia as has been found in organ cultures by other workers (Lasnitzki, 1956 ; Franks, 1961). This may be partly a question of the period spent in culture as well as the actual carcinogen chosen, but it clearly shows that absence of such a reaction in culture cannot be held to demonstrate the absence of carcinogenic properties of any material which has been added to the culture medium. The present experiment does not throw a direct light on the converse question, namely whether the presence of hyperplasia or metaplasia *in vitro* indicates that a chemical which has been added to the medium is in fact carcinogenic, which is the basis for some suggested tests for chemical carcinogenicity. It is significant in this regard, however, that the implants examined in the earlier stages showed metaplasia and functional overactivity (in the form of mucous and keratinised cysts) as frequently in the control as in the carcinogen-treated implants. Bronchial hyperplasia later on was more common in the carcinogen-treated implants but not exclusively so even then. It is in fact only when adenomata arise that any clearcut distinction can be made between the two groups, and the inference is clearly that the detection of carcinogenic activity under these conditions still depends on the observation of the formation of actual tumours.

The present experiments throw some light on the role of the host on the development of the tumours after the primary initiation *in vitro*. It has been suggested that an important part in the process is due to primary action of the carcinogenic stimulus on tissues at a distance from the site of tumour formation, e.g. the lymphoid tissues. In the present experiments such stimulus or effect was lacking; the tissues around the site of implantation showed no changes suggestive of the presence of even small quantities of carcinogen. It seems unlikely, therefore, that free carcinogen at any distant site played any part in the tumour production. It is of course not possible to exclude the participation of other tissues in a reaction to changes already induced by the carcinogen, such as the alteration of the antigenic make-up of the implanted tissue as has been postulated by Green (1954).

The tissues involved in the implants include the lower part of the trachea and the whole of the lung and bronchial tree. It is noteworthy that the adenomata have been found in relation to the main bronchi, with some cartilage in the wall. This distribution is reminiscent of that found in the common lung cancer in man. In the present case, however, the carcinogen is presented equally to all parts of the tissue and there is no question of clearing mechanisms, such as ciliary action, being at work. The present results suggest that this part of the bronchial tree, for whatever reason, shows a particular sensitivity to the action of the chemical carcinogen.

SUMMARY

Explants derived from the lungs of late embryo and one month old inbred mice were cultured *in vitro* for eight days with and without chemical carcinogen in the medium. They were then implanted subcutaneously into adult mice of the same strain.

Subsequent examination, after killing these animals at intervals, showed the occurrence of lung adenomata in a high percentage of carcinogen treated implants after a number of months. Normal implants showed non-specific changes, in particular hyperplasia of lymphoid tissue, but no adenomata.

### REFERENCES

Berenblum, I. and Shubik, P.—(1947) *Br. J. Cancer*, **1**, 383.
Berwald, Y. and Sachs, L.—(1965) *J. natn. Cancer Inst.*, **35**, 641.
Earle, W. R.—(1943) *J. natn. Cancer Inst.*, **4**, 165.
Franks, L. M.—(1961) *Expl Cell Res.*, **22**, 56.
Green, H. N.—(1954) *Br. med. J.*, ii, 1374.
Lasnitzki, I.—(1956) *Br. J. Cancer*, **10**, 510.
Trowell, O. A.—(1954) *Expl Cell Res.*, **6**, 246.